# The Overlap of Lung Tissue Transcriptome of Smoke Exposed Mice with Human Smoking and COPD

**DOI:** 10.1038/s41598-018-30313-z

**Published:** 2018-08-08

**Authors:** Ma’en Obeidat, Anna Dvorkin-Gheva, Xuan Li, Yohan Bossé, Corry-Anke Brandsma, David C. Nickle, Philip M. Hansbro, Rosa Faner, Alvar Agusti, Peter D. Paré, Martin R. Stampfli, Don D. Sin

**Affiliations:** 1 The University of British Columbia Center for Heart Lung Innovation, St Paul’s Hospital, Vancouver, Canada; 2 Department of Pathology and Molecular Medicine, McMaster Immunology Research Centre, Hamilton, ON Canada; 3 Institut universitaire de cardiologie et de pneumologie de Québec, Québec, Canada; 4 Department of Molecular Medicine, Laval University, Québec, Canada; 5 University of Groningen, University Medical Center Groningen, Department of Pathology and Medical Biology, Groningen, The Netherlands; 6 Merck & Co., Inc., MRL, Kenilworth, New Jersey United States of America; 7 Priority Research Centre for Healthy Lungs, University of Newcastle, Newcastle, New South Wales Australia; 8 Hunter Medical Research Institute, Newcastle, New South Wales Australia; 9 Fundacio Clinic per a la Recerca Biomedica Barcelona, Barcelona, Spain; 10 Respiratory Institute, Hospital Clinic, University of Barcelona, Institut d’investigacions Biomèdiques August Pi i Sunyer (IDIBAPS), Barcelona, Spain; 11 Respiratory Division, Department of Medicine, University of British Columbia, Vancouver, BC Canada; 12 Department of Medicine, Firestone Institute of Respiratory Health at St. Joseph’s Healthcare, McMaster University, Hamilton, ON Canada

## Abstract

Genome-wide mRNA profiling in lung tissue from human and animal models can provide novel insights into the pathogenesis of chronic obstructive pulmonary disease (COPD). While 6 months of smoke exposure are widely used, shorter durations were also reported. The overlap of short term and long-term smoke exposure in mice is currently not well understood, and their representation of the human condition is uncertain. Lung tissue gene expression profiles of six murine smoking experiments (n = 48) were obtained from the Gene Expression Omnibus (GEO) and analyzed to identify the murine smoking signature. The “human smoking” gene signature containing 386 genes was previously published in the lung eQTL study (n = 1,111). A signature of mild COPD containing 7 genes was also identified in the same study. The lung tissue gene signature of “severe COPD” (n = 70) contained 4,071 genes and was previously published. We detected 3,723 differentially expressed genes in the 6 month-exposure mice datasets (FDR <0.1). Of those, 184 genes (representing 48% of human smoking) and 1,003 (representing 27% of human COPD) were shared with the human smoking-related genes and the COPD severity-related genes, respectively. There was 4-fold over-representation of human and murine smoking-related genes (P = 6.7 × 10^−26^) and a 1.4 fold in the severe COPD -related genes (P = 2.3 × 10^−12^). There was no significant enrichment of the mice and human smoking-related genes in mild COPD signature. These data suggest that murine smoke models are strongly representative of molecular processes of human smoking but less of COPD.

## Introduction

Chronic obstructive pulmonary disease (COPD) affects 300 million people and is currently the third leading cause of death worldwide^1^. Although the exact mechanisms of pathophysiology are unknown, it is widely accepted that COPD is under genetic and environmental control with cigarette smoking being the most important modifiable risk factor in the Western world^2^.

Recent advances in genomics have enabled genome-wide mRNA profiling to gain novel insights into COPD pathogenesis^3–8^. While these studies report genes associated with disease phenotypes or smoking, follow up *in vitro* and *in vivo* studies are required to disentangle mechanism and establish causality. Of the *in vivo* models, mice are commonly used to determine the effects of cigarette smoking in the pathogenesis of COPD (reviewed in references^9–15^). Generally, 6 months of smoke exposure is used to induce histological and functional abnormalities in murine lungs that mimic those of human disease including emphysema, airway remodeling and pulmonary hypertension, though the changes are relatively mild compared with those observed in long-term human smokers^16^. However, more recent methods can replicate these features as well as the impairment of lung function in 8 weeks with nose-only exposure^17,18^. Shorter exposure times are generally used to model inflammatory mechanisms^11,19^. How gene expression profiles compare between short term and long term smoke exposure is currently not well understood. Moreover, although mice are commonly employed to model COPD, the extent to which murine experiments mimic the human condition is uncertain.

The availability of genome-wide transcriptomic signatures in lung tissue enables comparisons between human and murine models following short- and long-term cigarette smoke exposures. The aims of this study were to compare and contrast the molecular changes in murine models following short and long term exposures with the molecular changes in human lungs induced by cigarette smoke. Most importantly, we sought to determine, if the human “COPD” lung tissue gene expression signature is captured in murine lungs exposed to cigarette smoke.

## Results

### Murine gene expression signatures following short-term smoke exposure

The six murine studies involving short term smoke exposure are summarized in Table 1.

**Table 1:** The 6 murine studies used to detect smoking gene signature.

GEO accession and reference	Exposure duration (weeks)	Strain	Gender	Age (weeks)	Samples (RA/CS)	TPM (µg/L)	Cigarette
GSE33561	6–7	AKR/J	M	6–8	2/3	90	2R4F
GSE33512	16	C57BL/6	M	12	4/4	100–120	1R3F
GSE52509	16	C57BL/6	F	8–10	3/3	500	3R4F
GSE17737	12	C57BL/6	F	12	5 FA/5	NA	NA
GSE55127	8	BALB/C	F	6–8	4/5	>600	3R4F*
GSE18344	8	CD-1	F	13	4/4	750	2R4F
GSE52509	24	C57BL/6	F	24	3/3	500	3R4F
GSE17737	24	C57BL/6	F	24	6 FA/6	NA	NA

RA: room air; FA: forced air; CS: cigarette smoke; *filters removed.

**Figure 1:**
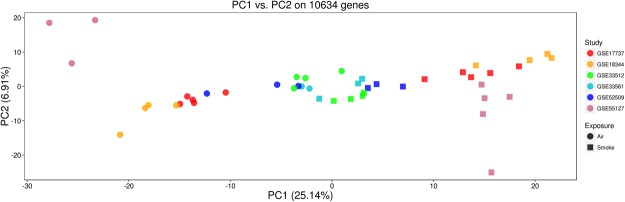
Principal component analysis of the 6 short-term exposure murine studies used to detect smoking gene signature.

Principal component analysis (PCA) performed on the 10,634 common genes led to the exclusion of one sample of air exposed mice from study GSE55127, which was a clear outlier. The resulting PCA plot shows that the 6 studies were homogenous in terms of expression changes and demonstrated clustering based on smoke-exposure status (Fig. 1).

Gene expression analysis of the pooled dataset from the 6 murine studies identified 3,723 genes that were differentially expressed at an FDR cutoff of 10%. Of the differentially expressed genes, 3,519 genes had 3,687 human gene orthologs. The use of a more stringent FDR cutoff of 5% or 1% reduced the number of differentially expressed genes to 3,051 and 2,021, respectively. The most significant differentially expressed genes were cxcl1 (C-X-C motif chemokine ligand 1), gpnmb (glycoprotein nmb) and cd84 (Table 2).

**Table 2:** The top 20 smoking related genes in the murine lungs.

Short term (8–16 week) smoke exposure				24 week smoke exposure			
Gene	logFC	P.Value	FDR	Gene	logFC	P.Value	FDR
Cxcl1	2.24	1.41E-16	1.50E-12	Zranb3	2.52	8.71E-19	9.21E-15
Gpnmb	2.04	5.80E-15	3.08E-11	Pld3	1.23	8.27E-17	4.37E-13
Cd84	0.96	1.51E-14	5.35E-11	Noxo1	1.84	6.08E-16	2.14E-12
Cd68	1.61	2.18E-14	5.80E-11	Ctsk	2.69	9.76E-16	2.58E-12
Slc7a11	1.90	3.45E-14	7.34E-11	Lhfpl2	1.68	1.30E-15	2.76E-12
Gdf15	1.25	4.44E-14	7.87E-11	Saa3	4.13	2.80E-15	4.93E-12
Tnfaip2	0.66	6.33E-14	9.61E-11	Lgals3	1.00	4.43E-15	5.04E-12
Ccl3	2.23	1.07E-13	1.42E-10	Mmp12	3.77	4.56E-15	5.04E-12
Asgr1	−1.02	1.84E-13	2.11E-10	Clec4n	1.77	4.61E-15	5.04E-12
Zranb3	1.84	1.98E-13	2.11E-10	Lrp12	1.35	4.77E-15	5.04E-12
Ctsk	2.00	2.25E-13	2.17E-10	Itih4	1.64	1.03E-14	9.87E-12
Lgals3	1.11	2.53E-13	2.17E-10	Cstb	0.69	1.41E-14	1.25E-11
Saa3	3.74	2.66E-13	2.17E-10	Ccl9	1.55	1.53E-14	1.25E-11
Myo5a	1.01	4.07E-13	2.94E-10	Ctsz	1.09	2.76E-14	2.08E-11
Cxcl5	2.92	4.15E-13	2.94E-10	Zmynd15	1.40	3.48E-14	2.45E-11
Cstb	0.70	5.30E-13	3.52E-10	Cd68	1.75	4.27E-14	2.78E-11
Lhfpl2	1.20	7.04E-13	4.04E-10	Npy	2.13	4.47E-14	2.78E-11
Cyp1b1	2.43	7.16E-13	4.04E-10	Lgmn	0.79	5.20E-14	3.06E-11
Hmox1	0.80	7.22E-13	4.04E-10	Gpnmb	2.39	1.21E-13	6.72E-11
Mmp12	3.29	1.14E-12	6.04E-10	Marco	2.29	1.66E-13	8.79E-11

LogFC: log fold change. FDR: false discovery rate.

### Murine gene expression signatures following long-term smoke exposure

The gene expression analysis in the pooled dataset from the two murine studies which involved 6 months smoke exposure identified 3,106 genes that were differentially expressed at an FDR cutoff of 10%. Of these, 2,989 genes had 3,116 human gene orthologs. Table 2 shows the top 20 differentially expressed genes in the short and the 6 month smoke exposure experiments.

### Comparison of human versus murine lung gene expression profiles related to cigarette smoke exposure or smoking status

**Figure 2:**
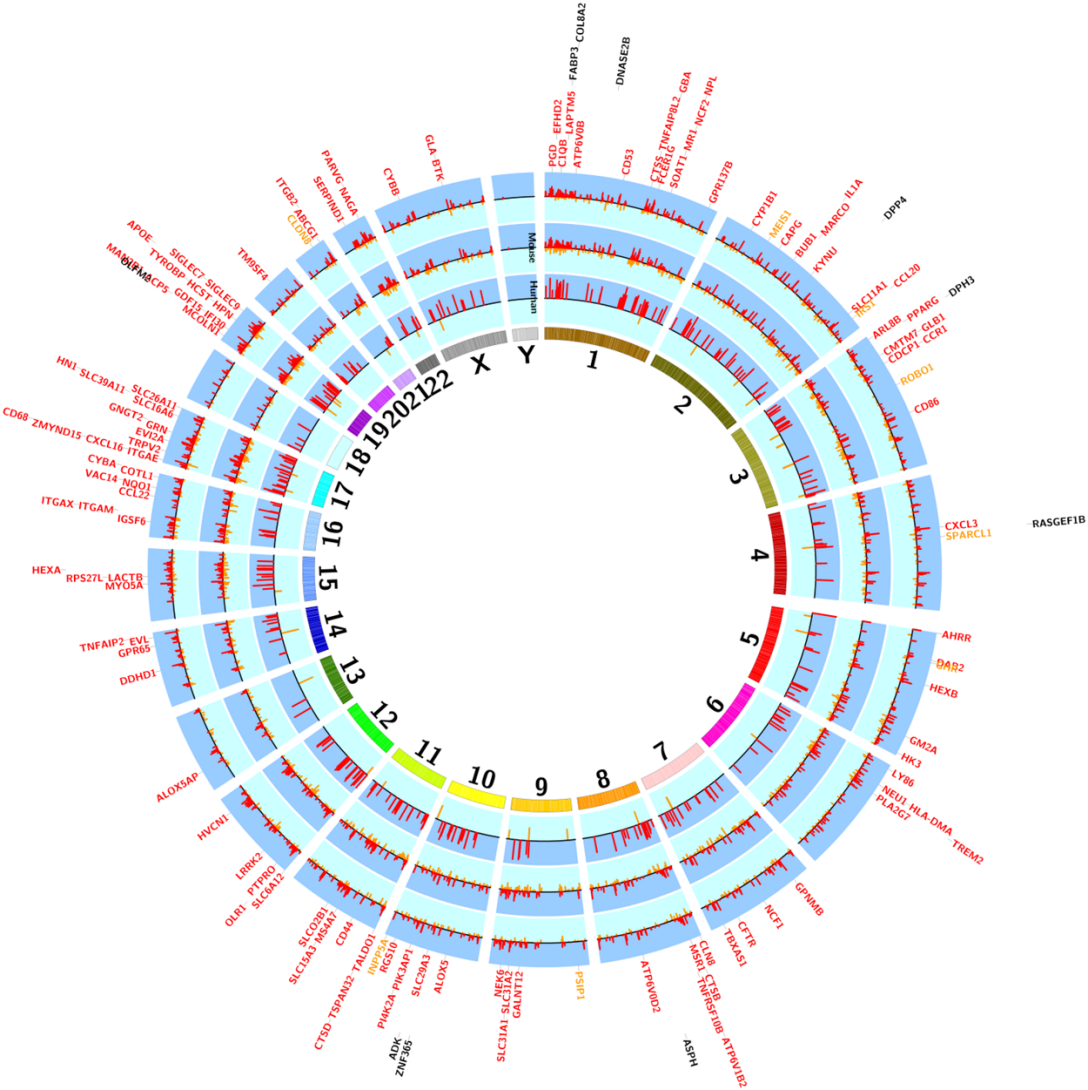
Circos plot of smoking related genes overlapping between human and murine lungs. Genes are shown based on their chromosomal positions (in the human genome) in the outer most circle. The first circle from the inside represents genes from the short-term smoke exposed mouse while the second circle represents genes from the long (24 weeks) term smoke-exposed mouse and the outer most circle represent the human smoking-related genes. Each line represents a gene: inward lines labeled in orange represent down-regulated genes while outward lines in red represent up-regulated genes. Gene symbols are colored accordingly with down and up-regulated genes depicted as orange and red, respectively. The length of the line is proportional to the –log10 p values for differential expression in human and for the –log10 FDR values in murine data. Gene symbols in black are genes that showed opposite direction of effect between mice and humans.

We next sought to evaluate the extent of overlap between murine and human smoking signatures. A total of 184 genes, representing 48% of human smoking signature genes, were shared between the human and short-term mice smoking exposure. Of those, 148 and 14 genes were up- and down-regulated in both datasets, respectively and 22 had an opposite direction of effect between the two datasets. A circos plot comparing the human lung tissue smoking signature of 386 genes (current vs. never smokers) with the 3,687 genes related to short term exposure in mice is shown in Fig. 2. When compared to the long-term exposure murine models (6 months), 168 genes demonstrated overlap with the human signature and of these, 146 and 9 genes were up- and down-regulated in both datasets, respectively and 13 had an opposite direction of effect. Comparing the human smoking signature to both short and long-term smoking exposure in mice, 139 genes overlapped in all three studies, and of these 121 and 8 genes were up and down-regulated in all three studies and 10 genes had an opposite direction of effect in the human dataset. A list of the top 20 (based on the P values in the human data) overlapping genes is shown in Table 3. The list of overlapping genes included aryl-hydrocarbon receptor repressor (AHRR), CYP1B1 cytochrome P450 family 1 subfamily B member 1 (CYP1B1), C-X-C motif chemokine ligand 16 (CXCL16), NAD(P)H quinone dehydrogenase 1 (NQO1) and serpin family D member 1 (SERPIND1).

**Table 3:** Top 20 smoking-related genes overlapping between human and murine lungs.

Gene	Mouse short-term logFC	Mouse short-term P value	Mouse short-term FDR	Mouse long-term logFC	Mouse long-term P value	Mouse long-term FDR	Human logFC	Human P value
AHRR	1.81	4.67E-10	5.71E-08	1.27	1.54E-07	6.43E-06	2.61	3.28E-20
CYP1B1	2.43	7.16E-13	4.04E-10	0.89	9.33E-11	1.28E-08	2.00	6.12E-20
CXCL16	0.32	4.03E-05	4.55E-04	0.65	7.55E-10	7.83E-08	0.75	8.74E-18
NQO1	1.60	9.10E-09	6.13E-07	0.30	3.52E-06	7.90E-05	0.93	1.17E-17
SERPIND1	0.50	1.86E-07	6.37E-06	0.76	1.08E-08	7.24E-07	3.73	5.20E-17
PGD	0.56	1.38E-11	3.54E-09	0.25	8.56E-05	1.04E-03	0.72	6.37E-17
NEK6	0.57	9.69E-10	9.81E-08	0.71	5.95E-08	2.94E-06	1.02	8.47E-17
SLC31A1	0.15	8.04E-04	5.11E-03	0.13	3.00E-03	1.77E-02	1.02	1.15E-16
ALOX5AP	0.52	1.42E-07	5.08E-06	0.49	2.06E-07	8.11E-06	0.65	3.07E-16
TREM2	1.26	9.92E-11	1.66E-08	1.50	8.80E-08	4.05E-06	1.74	4.39E-16
COL8A2	−0.30	6.87E-06	1.12E-04	−0.23	1.71E-03	1.14E-02	1.00	5.82E-16
OLR1	0.97	4.97E-08	2.19E-06	0.89	1.62E-06	4.17E-05	0.60	6.27E-16
ZNF365	−0.22	9.04E-03	3.48E-02	−0.28	1.52E-05	2.64E-04	1.63	6.52E-16
ATP6V0D2	1.24	3.48E-10	4.56E-08	1.04	1.11E-06	3.18E-05	2.56	7.64E-16
NCF2	0.55	4.87E-07	1.37E-05	0.52	4.29E-07	1.47E-05	0.74	1.13E-15
ACP5	0.88	1.43E-08	8.33E-07	1.11	1.14E-05	2.10E-04	1.12	1.46E-15
CYBB	0.60	1.40E-05	1.96E-04	0.61	5.37E-05	7.16E-04	1.22	2.08E-15
DNASE2B	−0.29	7.95E-04	5.06E-03	−0.37	3.95E-04	3.56E-03	2.11	2.56E-15
GM2A	0.10	2.34E-02	7.40E-02	0.16	8.51E-04	6.60E-03	0.78	5.31E-15
GNGT2	0.44	1.53E-04	1.34E-03	0.19	2.19E-02	7.97E-02	0.87	5.31E-15

The 139 overlapping genes were enriched in numerous gene ontology processes related to defense and immune response, glycosphingolipid and ceramide catabolic processes (Table 4).

**Table 4:** Gene ontology processes enriched in human and murine smoking overlapping genes.

Gene ontology (GO) pathway	P value	FDR
**The 139 genes overlapping the mice and human smoking signatures**		
Immune response	2.20E-11	9.89E-09
Defense response	1.15E-11	9.89E-09
Glycosphingolipid catabolic process	1.78E-10	5.33E-08
Glycolipid catabolic process	3.53E-10	7.93E-08
Immune system process	8.30E-10	1.49E-07
Inflammatory response	1.23E-09	1.58E-07
Phagosome maturation	1.08E-09	1.58E-07
Ceramide catabolic process	6.79E-09	7.63E-07
Response to stimulus	8.71E-09	8.70E-07
Sphingolipid catabolic process	1.94E-08	1.74E-06
Response to chemical stimulus	2.17E-08	1.77E-06
Membrane lipid catabolic process	2.65E-08	1.99E-06
Glycosphingolipid metabolic process	5.50E-08	3.80E-06
Lipid storage	2.43E-07	1.56E-05
Antigen processing and presentation of peptide antigen	6.98E-07	4.18E-05
**The 48 genes overlapping smoking in mice and human and COPD signatures**		
Antigen processing and presentation of peptide antigen via MHC class I	2.00E-04	2.85E-02
Pyridine nucleotide metabolic process	4.00E-04	2.85E-02
Catabolic process	4.00E-04	2.85E-02
Nicotinamide nucleotide metabolic process	4.00E-04	2.85E-02
Organic substance catabolic process	6.00E-04	2.85E-02
Pyridine-containing compound metabolic process	6.00E-04	2.85E-02
Transmembrane transport	7.00E-04	2.85E-02
Oxidoreduction coenzyme metabolic process	7.00E-04	2.85E-02
Carbohydrate catabolic process	8.00E-04	2.90E-02
Carbohydrate derivative catabolic process	9.00E-04	2.93E-02
Glucose catabolic process	1.50E-03	4.35E-02
Antigen processing and presentation of exogenous peptideantigen via MHC class I	1.60E-03	4.35E-02

### Comparison of murine and human smoking signature with COPD lung-tissue signature

**Figure 3:**
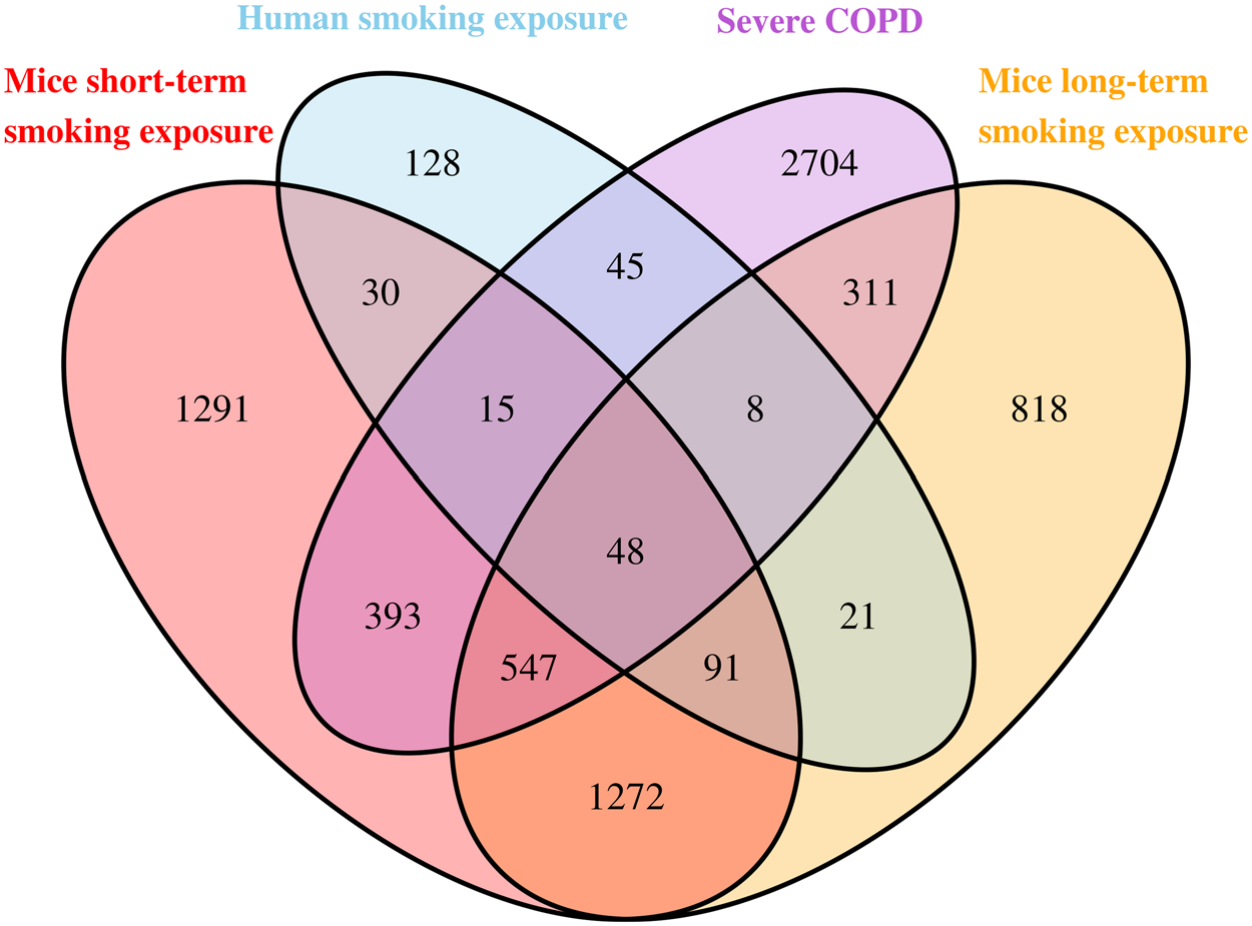
Overlap of severe COPD signature with human and murine smoking signatures.

To gain insights into the translational potential of the smoking gene signature, we tested for overlap with published human COPD signatures in lung tissue. The Faner *et al*. dataset included 70 former smokers with COPD from GOLD grades 1 to 4^4^. Using this dataset we identified 4,071 genes that were differentially expressed between patients in GOLD 3/4 vs. GOLD 1/2. A total of 1,003 “smoking” genes (27%) from the short-term murine smoking experiments overlapped with the “severe COPD signature” from the Faner *et al*. Comparison of the human smoking and COPD signature showed that 116 “smoking” genes (30%) from the lung eQTL dataset overlapped with the “severe COPD signature” of Faner *et al*. (Fig. 3). Of the 3,116 “smoking” genes derived from the 6 month exposure model in mice, 1,958 (53%) overlapped with the “smoking” genes derived from the short-term smoke exposure in mice and 168 genes (44%) overlapped with the human smoking signature from the eQTL study, and 914 genes (22%) overlapped with the “severe COPD” signature in the Faner *et al*. study.

Overall, 48 genes were common to both smoking signatures in mice (short and long term exposure) as well as the human smoking and severe COPD signatures (Supplementary Table 1). All of these genes except one showed the same direction of effect across studies i.e. up-regulated in smoking and in COPD or vice versa. These 48 genes were enriched in a number of gene ontology processes that are summarized in Table 4 including antigen processing and presentation, pyridine and nicotinamide nucleotide metabolic process, catabolic processes, transmembrane transport, oxidoreduction coenzyme metabolic process, and carbohydrate and glucose catabolic processes.

An additional relevant question to this work was whether or not smoking signature of mice and human will show enrichment in mild COPD as opposed to severe COPD signature. To answer this question, we analyzed the transcriptome of lung tissue eQTL study comparing mild COPD cases to controls. At and FDR <0.1 cutoff, this analysis identified 7 genes differentially expressed between mild COPD cases and controls (Supplementary Table 2).

To quantify the extent of overlap among the different studies, we used a Fisher’s exact test to determine whether there was significant enrichment of the human smoking or disease signatures in murine smoking signatures (Table 5). Differentially expressed genes from all the studies showed an over-representation in the mice data. The strongest enrichment was observed between the short and long-term mice smoking signatures (5.5 fold enrichment, p = 1.6 × 10^−309^). The results also showed almost 4-fold enrichment of human smoking genes in the mice smoking signature (P = 6.7 × 10^−26^). Of the lung tissue disease signatures, the severe COPD signature from the study of Faner *et al*. was over-represented in the short term murine smoking signature (1.2 fold enrichment, P = 4.2 × 10^−05^) and was also over-represented in the 6 month smoking models (1.4 fold, p = 2.3 × 10^−12^). Interestingly though, the mild COPD signature was not over-represented in the mice or human smoking signatures.

**Table 5:** Enrichment of human smoking and disease signatures in the mice smoking signature.

Study	Short-term mouse smoking signature	Human Smoking signature (Bossé *et al*.)	Severe COPD Signature (Faner *et al*.)	6 months mouse smoking signature	Mild COPD signature
Short-term smoking signature	NA	**3.8** (**p** = **6**.**7** × **10**^**−26**^)	**1.2** (**p** = **4**.**2** × **10**^**−5**^)	**5**.**5** (**p** = **1**.**6** × **10**^**−309**^)	3.5 (p = 0.3)
Human Smoking signature (Bossé *et al*.)	3.8 (**p** = **6**.**7** × **10**^**−26**^)	NA	1.4 (**p** = **3**.**5** × **10**^**−3**^)	**3**.**8** (**p** = **1**.**6** × **10**^**−26**^)	0* (p = 1)
Severe COPD Signature (Faner *et al*.)	**1.2** (**p** = **4**.**2** × **10**^**−5**^)	**1.4** (**p** = **3**.**5** × **10**^**−3**^)	NA	**1**.**4** (**p** = **2**.**3** × **10**^**−12**^)	1.6 (p = 0.63)
6 months mouse smoking signature	**5**.**5** (**p** = **1**.**6** × **10**^**−309**^)	**3**.**8** (**p** = **1**.**6** × **10**^**−26**^)	**1**.**4** (**p** = **2**.**3** × **10**^**−12**^)	NA	4.6 (p = 0.22)
Mild COPD signature	3.5 (p = 0.3)	0* (p = 1)	1.6 (p = 0.63)	4.6 (p = 0.22)	NA

Each cell shows the enrichment fold and the P value associated with it for these two studies. *Indicates that there were no overlapping genes between mild COPD and human smoking.

### Integrative genomics of smoking related genes common to both human and murine lungs

To extend the gene expression findings to large scale human genetic studies of lung function we investigated whether any of the genes whose expression was related to smoking in both human and murine lungs were under genetic control in human lung tissue (i.e. were lung expression quantitative trait loci [eQTLs]). We found that 60 of the 139 genes have significant eQTLs (10% FDR) with a total of 7834 eQTLs.

Next, we restricted the analysis to the most significant eQTL per probeset (based on the eQTL p values) which led to a final SNP list of 73 (some SNPs were top eQTLs for more than one probeset). The 73 eQTLs were tested for associations with lung function in publically available large-scale genome-wide association studies (GWASs) datasets: SpiroMeta^20^ and the UKBilEVE studies^21^. The results for SNPs with p < 0.05 are shown in Supplementary Table 3 for eQTLs that had p value < 0.05 for association with lung function in SpiroMeta or UKBiLEVE. The only SNP that was associated with lung function at FDR < 0.05 was rs1081512, which was an eQTL for CTSS (cathepsin S) gene. It was also strongly associated with FEV_1_ in the SpiroMeta GWAS (P = 6.07E-05, FDR = 0.004).

## Discussion

Pre-clinical animal models represent a valuable tool for understanding the pathogenesis of COPD and identifying novel therapeutics and biomarkers. However, to date, there has been a scarcity of data that have directly compared molecular profiles in the lungs of smoke-exposed mice that have been used to model COPD against those of human lungs in order to determine how (and if) ‘disease’ in these animals is representative of the human condition. A recent study by Yun *et al*. reported the overlap of mice and human smoking signatures and identified many overlapping genes, but very few that were shared with COPD signature^22^. Earlier, Morissette *et al*. also investigated the overlap of genes differentially affected by smoking in both mice and human lung tissues^8^. They found an enrichment of genes that were significantly modulated by cigarette smoke in humans and in mice, and that the majority of biological functions modulated by cigarette smoke in humans were also affected in mice^8^. Both studies, however, did not compare short vs. long term smoke exposures of mice and did not identify genetic variants relating to the expression of genes of interest.

By directly comparing and contrasting the gene expression profiles of smoke-exposed (both long and short-term) murine lungs against a large number of human lungs of current and ex smokers across the full spectrum of COPD severity (and also versus former smokers), we have made several important observations. They include: (1) the identification of overlapping 3,723 and 3,106 genes that were differentially expressed in short and long-term smoke exposure in murine lungs, respectively (5.5-fold enrichment of short term signature in the long-term signature, P = 1.6 × 10^−309^), suggesting that acute transcriptomic changes in the lungs related to cigarette smoking are largely retained over longer term, when morphologic appearances of emphysema, airway remodeling and mild pulmonary hypertension become measurable in mice; (2) a significant overlap of genes in smoke-exposed murine lungs (48% from short-term exposure and 44% from long-term exposures) with those of human lungs explanted from current smokers. There was a 3.8 fold enrichment of the human “smoking” lung signature in the murine lungs (p = 1.6–6.7 × 10^−26^); and (3) a 1.4 fold enrichment of severe COPD gene expression signature in the human “smoking” lung signature (p = 3.5 × 10^−3^), with a 1.2 fold enrichment in short-term smoke- exposed murine (p = 4.2 × 10^−5^) and a 1.4 fold enrichment in long-term smoke-exposed murine lungs (p = 2.3 × 10^−12^). There were 48 genes that were common to the lungs of both smoked-exposed mice and current smokers and severe COPD, suggesting that the long term smoking exposure of mice results in transcriptomic changes that are also found in severe COPD patients even following smoking cessation. Of these the association of the gene encoding for cathepsin S was also supported in large scale human genetics studies of lung function. Finally, the murine and human smoking signatures were not over-represented in mild COPD signature, suggesting that overall mice models are better representative of smoking but less so of COPD in humans.

The smoking genes that overlapped between murine and human lung tissue included aryl hydrocarbon receptor repressor (AHRR), cytochrome P450 family 1 subfamily B member 1 (CYP1B1), C-X-C motif chemokine ligand 16 (CXCL16), NAD(P)H quinone dehydrogenase 1 (NQO1) and serpin family D member 1 (SERPIND1), all of which were up-regulated in the lung tissues of smokers. The AHRR gene has been well studied and a large number of publications have reported changes in its methylation and expression related to smoking^23,24^. AHRR encodes a ligand-activated transcription factor that inhibits the aryl hydrocarbon receptor pathway, which, in turn, increases the expression of xenobiotic-metabolizing enzymes that break down environmental pollutants, such as polycyclic aromatic hydrocarbons contained in cigarette smoke^25^. CYP1B1 is a phase I enzyme that is involved in the conversion of procarcinogens in cigarette smoke to carcinogenic intermediates^26^. The expression of CYP1B1 was found to be up-regulated in a number of tissues including the lungs following cigarette smoke exposure^27^. NQO1 is an enzyme involved in the detoxification of mutagenic and carcinogenic quinones, by preventing electron transfer and the generation of free radicals and reactive oxygen species^28^ and converting the intermediates to the less toxic hydroquinones^29^. SERPIND1 encodes the heparin cofactor II (HCII), which is an endogenous thrombin inhibitor that protects against vascular remodeling and atherosclerosis via its inhibition of thrombin in the vascular wall^30^. It may also play a role in enhancing cell motility and promoting metastasis in non-small cell lung cancer^31^.

Almost half (48%) of genes making up the human smoking signature overlapped with those differentially expressed in the murine smoked lung. The overlapping genes were enriched in processes related to host defense and immune responses including those that involve glycosphingolipid and ceramide catabolic pathways. These processes are well known to be affected by smoking^32–34^. The significant enrichment of human smoking signatures in the murine lung following short and long-term smoke exposure suggests that mice models of smoking do, in fact, reflect molecular changes that occur with smoking in humans. There are some caveats, however. For instance, we found that for ~6% of the human “smoking” lung genes the change in gene expression in the murine lungs was in the opposite direction. This may be due to different molecular responses to smoking between human and mice lungs. Alternatively, it may reflect the duration of cigarette smoke exposure between humans and mice. The duration of smoke exposure for mice ranged from 6–24 weeks compared to years of smoking in humans. However, this can be considered representative in a mouse that have an average life span of 1.5–2 years.

Using integrative genomics we showed that 43% of the overlapping smoking signature genes were under genetic control in lung tissue. The SNP that showed the strongest association with lung function in large scale genetic studies was an eQTL for the cathepsin S gene (CTSS). The CTSS gene encodes an elastin-degrading proteinase which is highly expressed by macrophages and dendritic cells^35^ and plays an active role in adaptive immune responses^36^. The major inhibitor of cathepsin S is cystatin C which was recently identified as a COPD causal gene using an integrative genomics approach^37^.

Our current study has some limitations. First, the sample sizes for the studies included may have led to false negative results. Second, the unit of analysis in this study was gene expression, yet translation and post translational modifications of proteins in lung tissue may also be similar or different between mice and human and between smokers with and without COPD. Third, mice have different pathophysiology compared to humans. For example, studies have shown that in humans, the loss of small airways proceeds the development of emphysema before COPD is detectable with spirometry^38^. Finally, the cellular heterogeneity of murine and human lung tissue samples may have limited our ability to detect overlapping signatures.

In conclusion, the current study uncovered a strong similarity between short and long term smoking effects on lung transcriptome in mice and a strong overlap with the human smoking signature. The study additionally uncovered genes common to smoking and COPD signatures in mice and humans which warrants further study.

## Methods

### Data sources

#### Human Lung tissue eQTL and smoking signature study

To compare murine lung smoke exposure induced gene expression against human smoking gene expression signatures, we used a large human dataset that has been previously described. The lung expression quantitative trait loci study (eQTLs) profiled 1,111 human lung tissue from current and ex-smokers and non-smokers^39–42^. Briefly, non-tumour lung specimens were collected from patients undergoing lung surgery at three different sites: Institut Universitaire de Cardiologie et de Pneumologie de Québec (IUCPQ), Laval University (Quebec, Canada), University of British Columbia (UBC, Vancouver, Canada) and University of Groningen (Groningen, the Netherlands. Gene expression profiling was performed using an Affymetrix custom array (GPL10379), which contained 51,627 non-control probesets and data were normalized using RMA^43^. Genotyping was performed using the Illumina Human1M-Duo BeadChip array. Genotype imputation was undertaken using the 1000 G reference panel. Following standard microarray and genotyping quality controls, data from 1,111 patients were available including 409 from Laval, 339 from UBC and 363 from Groningen. Association testing for each variant with mRNA expression in either cis (within 1 Mb of transcript start site) or in trans (all other combinations) was undertaken separately for each study sample, after which the results were meta-analyzed using inverse variance weighting. A genome-wide 10% false discovery rate (FDR) was applied to this analysis. The smoking gene signature in the eQTL study has been previously published^7^ and consisted of 386 genes that were differentially expressed between current vs. never smokers (henceforth referred to as “human smoking signature”).

The lung eQTL study was also used to identify mild COPD signature. We performed differential expression analysis between mild COPD (FEV1 ≥80% predicted and FEV1/FVC <0.7) and controls (FEV1 ≥80% predicted and FEV1/FVC >0.7). The analysis was adjusted for age, sex and smoking status and the sample sizes were 58 mild COPD patients (12 from Laval and 46 from UBC) and 107 control subjects (11 from Laval and 96 from UBC). Results were combined using meta analysis using inverse variance weighting fixed effect model.

All methods were carried out in accordance with relevant guidelines and regulations. Study participants informed consent was obtained from all subjects, and data access and analyses protocols were approved by the University of British Columbia Office of Research Ethics.

#### Mouse gene expression data

Lung gene expression profiles of six publically available datasets (n = 48 samples) were obtained from the Gene Expression Omnibus (GEO) (accession numbers GSE33561, GSE33512, GSE52509, GSE17737, GSE55127, GSE18344)^44^. GSE33512, GSE55127, GSE52509 and GSE33561 datasets were pre-processed as described in the corresponding source publications. GSE17737 and GSE18344 datasets contained samples profiled on Affymetrix Mouse Genome 430 2.0 arrays. These arrays were normalised with frozen Robust Multi-array Analysis (fRMA), a procedure that allows microarrays to be pre-processed individually or in small batches and allows data to be combined into a single dataset for further analyses^45^. Since different profiling platforms contain different numbers of genes, we included 10, 634 genes in the analysis that were common to all platforms. A more detailed description is provided by Dvorkin-Gheva *et al*.^44^

To enable comparisons with smoking signatures from longer duration of smoking, we included samples from two additional GEO datasets (GSE52509 and GSE17737) that evaluated murine lung tissue expression changes following 24 weeks of smoking exposure. GSE52509 dataset was preprocessed as described in the corresponding publication, while the samples from GSE17737 were normalized with fRMA^44^. Samples from both datasets were combined and the technical variation was removed by using Distance-Weighted Discrimination (DWD) method^46^.

#### Differential gene expression analysis

We used the “limma” package^47^ to compare gene expression profiles of smoke-exposed mice from each dataset with those of control mice pooled across all experiments. T-statistics were followed by Benjamini–Hochberg adjustment for multiple testing^48^.

#### Lung tissue transcriptome signature of COPD severity

We used data from Faner *et al*. to determine which genes were differentially expressed across COPD disease severity^4^. Briefly, lung tissue samples were obtained from 70 former smokers with COPD who required thoracic surgery because of cancer or lung transplant. RNA samples were loaded onto an Affymetrix GeneChip Human Genome U219 Array Plate (Santa Clara, CA). The microarray data have been deposited in GEO (GSE69818)^4^. We identified 4,071 genes whose expression in lung tissue was different in patients with moderate or severe COPD (i.e. Global Initiative for Chronic Obstructive Lung Disease (GOLD) grades 3, 4) and those with mild COPD (defined by GOLD grades 1, 2). These differentially expressed genes will henceforth be referred to as “severe COPD signature”.

#### Overlap of murine and human genes

In order to compare results across murine and human studies, we restricted the analyses to murine genes that had a human ortholog using the BioMart-Ensembl database (release 88, March 2017 http://www.ensembl.org/info/about/publications.html). We retained only those human genes on chromosome 1 to 22, or on chromosome X or Y, based on the position information from the BioMart-Ensembl database.

#### Enrichment of gene signatures

A hypergeometric (Fisher’s exact) test was used to test for significant over or under-representation of common genes from two different studies.

## Electronic supplementary material

Supplementary Materials

## References

[1] WHO. (World Health Organization, 2014).

[2] Vestbo J (2013). Global strategy for the diagnosis, management, and prevention of chronic obstructive pulmonary disease: GOLD executive summary. Am J Respir Crit Care Med.

[3] Stepaniants S (2014). Genes related to emphysema are enriched for ubiquitination pathways. BMC Pulmonary Medicine.

[4] Faner, R. *et al*. Network analysis of lung transcriptomics reveals a distinct B-cell signature in emphysema. *Am J Respir Crit Care Med* **193**, 10.1164/rccm.201507-1311OC (2016). 10.1164/rccm.201507-1311OC 26735770

[5] Obeidat, M. *et al*. Integrative Genomics of Emphysema Associated Genes Reveals Potential Disease Biomarkers. *American journal of respiratory cell and molecular biology*, 10.1165/rcmb.2016-0284OC (2017). 10.1165/rcmb.2016-0284OC PMC5650084 28459279

[6] Campbell J (2012). A gene expression signature of emphysema-related lung destruction and its reversal by the tripeptide GHK. Genome medicine.

[7] Bosse Y (2012). Molecular signature of smoking in human lung tissues. Cancer research.

[8] Morissette MC (2014). Impact of cigarette smoke on the human and mouse lungs: a gene-expression comparison study. Plos one.

[9] Wright JL, Churg A (2010). Animal models of cigarette smoke-induced chronic obstructive pulmonary disease. Expert review of respiratory medicine.

[10] Churg A, Cosio M, Wright JL (2008). Mechanisms of cigarette smoke-induced COPD: insights from animal models. American journal of physiology. Lung cellular and molecular physiology.

[11] Stevenson CS, Birrell MA (2011). Moving towards a new generation of animal models for asthma and COPD with improved clinical relevance. Pharmacology & therapeutics.

[12] Vlahos R, Bozinovski S (2014). Recent advances in pre-clinical mouse models of COPD. Clinical science (London, England: 1979).

[13] Stevenson CS, Belvisi MG (2008). Preclinical animal models of asthma and chronic obstructive pulmonary disease. Expert review of respiratory medicine.

[14] Fricker M, Deane A, Hansbro PM (2014). Animal models of chronic obstructive pulmonary disease. Expert opinion on drug discovery.

[15] Jones B (2017). Animal models of COPD: What do they tell us?. Respirology (Carlton, Vic.).

[16] Andrew C, Don DS, Joanne LW (2011). Everything Prevents Emphysema. American journal of respiratory cell and molecular biology.

[17] Beckett EL (2013). A new short-term mouse model of chronic obstructive pulmonary disease identifies a role for mast cell tryptase in pathogenesis. The Journal of allergy and clinical immunology.

[18] Hsu AC (2015). Targeting PI3K-p110alpha Suppresses Influenza Virus Infection in Chronic Obstructive Pulmonary Disease. Am J Respir Crit Care Med.

[19] Vlahos R (2006). Differential protease, innate immunity, and NF-κB induction profiles during lung inflammation induced by subchronic cigarette smoke exposure in mice. American Journal of Physiology-Lung Cellular and Molecular Physiology.

[20] Artigas, M. S. *et al*. Sixteen new lung function signals identified through 1000 Genomes Project reference panel imputation. *Nature Communications* **6**, 8658, 10.1038/ncomms9658, http://www.nature.com/articles/ncomms9658#supplementary-information (2015). 10.1038/ncomms9658 PMC4686825 26635082

[21] Wain LV (2015). Novel insights into the genetics of smoking behaviour, lung function, and chronic obstructive pulmonary disease (UK BiLEVE): a genetic association study in UK Biobank. The Lancet Respiratory Medicine.

[22] Jeong HY (2017). Transcriptomic Analysis of Lung Tissue from Cigarette Smoke–Induced Emphysema Murine Models and Human Chronic Obstructive Pulmonary Disease Show Shared and Distinct Pathways. American journal of respiratory cell and molecular biology.

[23] Philibert R (2015). A quantitative epigenetic approach for the assessment of cigarette consumption. Frontiers in psychology.

[24] Reynolds, L. M. *et al*. Span hwp:id = “article-title-1” class = “article-title” DNA Methylation of the Aryl Hydrocarbon Receptor Repressor Associations With Cigarette Smoking and Subclinical Atherosclerosis span span hwp:id = “article-title-45” class = “sub-article-title” Clinical Perspective span. *Circulation: Cardiovascular Genetics* **8**, 707–716, 10.1161/circgenetics.115.001097 (2015). 10.1161/CIRCGENETICS.115.001097 PMC4618776 26307030

[25] Opitz CA (2011). An endogenous tumour-promoting ligand of the human aryl hydrocarbon receptor. Nature.

[26] Shimada T (1996). Activation of Chemically Diverse Procarcinogens by Human Cytochrome P-450 1B1. Cancer Research.

[27] Port JL (2004). Tobacco smoke induces CYP1B1 in the aerodigestive tract. Carcinogenesis.

[28] Schlager JJ, Powis G (1990). Cytosolic NAD(P)H:(quinone-acceptor)oxidoreductase in human normal and tumor tissue: effects of cigarette smoking and alcohol. Int J Cancer.

[29] Siegel D (2004). NAD(P)H:quinone oxidoreductase 1: role as a superoxide scavenger. Molecular pharmacology.

[30] Aihara K-i (2010). Heparin Cofactor II Attenuates Vascular Remodeling in Humans and Mice. Circulation Journal.

[31] Liao WY (2015). Heparin co-factor II enhances cell motility and promotes metastasis in non-small cell lung cancer. J Pathol.

[32] Stampfli MR, Anderson GP (2009). How cigarette smoke skews immune responses to promote infection, lung disease and cancer. Nature reviews. Immunology.

[33] Goldkorn, T., Chung, S. & Filosto, S. Lung cancer and lung injury: the dual role of ceramide. *Handbook of experimental pharmacology*, 93–113, 10.1007/978-3-7091-1511-4_5 (2013). 10.1007/978-3-7091-1511-4_5 PMC4370279 23563653

[34] Thatcher MO (2014). Ceramides mediate cigarette smoke-induced metabolic disruption in mice. American journal of physiology. Endocrinology and metabolism.

[35] Shi GP, Munger JS, Meara JP, Rich DH, Chapman HA (1992). Molecular cloning and expression of human alveolar macrophage cathepsin S, an elastinolytic cysteine protease. The Journal of biological chemistry.

[36] Riese RJ (1996). Essential role for cathepsin S in MHC class II-associated invariant chain processing and peptide loading. Immunity.

[37] Lamontagne, M. *et al*. Genetic regulation of gene expression in the lung identifies CST3 and CD22 as potential causal genes for airflow obstruction. *Thorax*, 10.1136/thoraxjnl-2014-205630 (2014). 10.1136/thoraxjnl-2014-205630 25182044

[38] Hogg JC (2004). The nature of small-airway obstruction in chronic obstructive pulmonary disease. N. Engl. J. Med..

[39] Lamontagne M (2013). Refining Susceptibility Loci of Chronic Obstructive Pulmonary Disease with Lung eqtls. Plos one.

[40] Hao K (2012). Lung eQTLs to Help Reveal the Molecular Underpinnings of Asthma. Plos Genetics.

[41] Obeidat ME (2013). GSTCD and INTS12 Regulation and Expression in the Human Lung. Plos one.

[42] Obeidat, M. E. *et al*. Molecular mechanisms underlying variations in lung function: a systems genetics analysis. *The Lancet Respiratory Medicine*, 10.1016/s2213-2600(15)00380-x (2015). 10.1016/S2213-2600(15)00380-X PMC5021067 26404118

[43] Irizarry RA (2003). Exploration, normalization, and summaries of high density oligonucleotide array probe level data. Biostatistics.

[44] Dvorkin-Gheva, A. *et al*. Total particulate matter concentration skews cigarette smoke’s gene expression profile. *ERJ Open Research* **2**, 10.1183/23120541.00029-2016 (2016). 10.1183/23120541.00029-2016 PMC5165723 27995131

[45] McCall MN, Bolstad BM, Irizarry RA (2010). Frozen robust multiarray analysis (fRMA). Biostatistics.

[46] Benito M (2004). Adjustment of systematic microarray data biases. Bioinformatics.

[47] Smyth, G. K. In *Bioinforma Comput Biol Solut Using R Bioconductor* (ed. Gentleman, R. *et al*.) (Springer, 2005).

[48] Benjamini, Y. & Hochberg, Y. Controlling the false discovery rate: a practical and powerful approach to multiple testing. *J R Stat Soc B* **57** (1995).

