# Supplementary material for: The Overlap of Lung Tissue Transcriptome of Smoke Exposed Mice with Human Smoking and COPD
Source: Sci Rep. 2018 Aug 8;8:11881. doi: 10.1038/s41598-018-30313-z (PMC6082828; doi:10.1038/s41598-018-30313-z)
Supplement: Supplementary file 1 — Supplementary Materials [file 41598_2018_30313_MOESM1_ESM.docx]

**The Overlap of Lung Tissue Transcriptome of Smoke Exposed Mice with Human Smoking and COPD**

**Supplementary Materials**

Ma’en Obeidat^1*^, Anna Dvorkin-Gheva^2*^, Xuan Li^1^, Yohan Bossé^3,4^, Corry-Anke Brandsma^5^, David C. Nickle^6^, Philip M. Hansbro^7,8^, Rosa Faner^9^, Alvar Agusti^9,10^, Peter D. Paré^1,11^, Martin R. Stampfli^2,12^, and Don D. Sin^1,11^

# Supplementary Tables

## Supplementary Table 1: List of 48 overlapping genes across mice and human smoking and COPD signatures.

| **Mouse gene** | **Human**  **gene** | **Mouse**  **logFC** | **Mouse**  **P value** | **Mouse**  **FDR** | **Mouse 24 weeks**  **logFC** | **Mouse 24 weeks**  **P value** | **Mouse 24 weeks**  **FDR** | **Human probeset ID** | **Human logFC** | **Human P value** | **COPD probeset ID** | **COPD**  **logFC** | **COPD**  **P value** | **COPD**  **FDR** | **Direction** |
| --- | --- | --- | --- | --- | --- | --- | --- | --- | --- | --- | --- | --- | --- | --- | --- |
| Pgd | PGD | 0.561 | 1.38E-11 | 3.54E-09 | 0.253 | 8.56E-05 | 1.04E-03 | 100138774_TGI_at | 0.723 | 6.37E-17 | 11746023_a_at | 0.385 | 1.07E-05 | 1.97E-03 | ++++ |
| Pgd | PGD | 0.561 | 1.38E-11 | 3.54E-09 | 0.253 | 8.56E-05 | 1.04E-03 | 100138774_TGI_at | 0.723 | 6.37E-17 | 11723205_a_at | 0.413 | 4.50E-05 | 4.36E-03 | ++++ |
| Pgd | PGD | 0.561 | 1.38E-11 | 3.54E-09 | 0.253 | 8.56E-05 | 1.04E-03 | 100305111_TGI_at | 0.671 | 8.82E-17 | 11746023_a_at | 0.385 | 1.07E-05 | 1.97E-03 | ++++ |
| Pgd | PGD | 0.561 | 1.38E-11 | 3.54E-09 | 0.253 | 8.56E-05 | 1.04E-03 | 100305111_TGI_at | 0.671 | 8.82E-17 | 11723205_a_at | 0.413 | 4.50E-05 | 4.36E-03 | ++++ |
| Cd53 | CD53 | 0.587 | 1.76E-11 | 4.07E-09 | 0.575 | 1.50E-06 | 3.93E-05 | 100133091_TGI_at | 0.344 | 2.08E-09 | 11716842_a_at | 0.276 | 9.02E-03 | 8.11E-02 | ++++ |
| Gpr137b | GPR137B | 0.444 | 8.25E-11 | 1.41E-08 | 0.511 | 4.69E-08 | 2.42E-06 | 100139575_TGI_at | 0.501 | 1.50E-12 | 11743011_a_at | 0.285 | 1.12E-02 | 9.15E-02 | ++++ |
| Fcer1g | FCER1G | 0.826 | 6.16E-10 | 6.97E-08 | 0.799 | 7.14E-12 | 1.68E-09 | 100160419_TGI_at | 0.367 | 7.51E-13 | 11718419_at | 0.270 | 7.92E-03 | 7.57E-02 | ++++ |
| Gba | GBA | 0.336 | 2.95E-09 | 2.34E-07 | 0.415 | 1.78E-07 | 7.25E-06 | 100305602_TGI_at | 0.424 | 4.04E-13 | 11751098_a_at | 0.221 | 7.35E-03 | 7.27E-02 | ++++ |
| Laptm5 | LAPTM5 | 0.447 | 4.30E-09 | 3.20E-07 | 0.509 | 3.14E-10 | 3.73E-08 | 100146335_TGI_at | 0.620 | 1.55E-14 | 11742922_at | 0.338 | 7.68E-05 | 5.74E-03 | ++++ |
| Laptm5 | LAPTM5 | 0.447 | 4.30E-09 | 3.20E-07 | 0.509 | 3.14E-10 | 3.73E-08 | 100146335_TGI_at | 0.620 | 1.55E-14 | 11742923_a_at | 0.277 | 7.20E-03 | 7.17E-02 | ++++ |
| Laptm5 | LAPTM5 | 0.447 | 4.30E-09 | 3.20E-07 | 0.509 | 3.14E-10 | 3.73E-08 | 100302489_TGI_at | 0.423 | 1.37E-14 | 11742922_at | 0.338 | 7.68E-05 | 5.74E-03 | ++++ |
| Laptm5 | LAPTM5 | 0.447 | 4.30E-09 | 3.20E-07 | 0.509 | 3.14E-10 | 3.73E-08 | 100302489_TGI_at | 0.423 | 1.37E-14 | 11742923_a_at | 0.277 | 7.20E-03 | 7.17E-02 | ++++ |
| C1qb | C1QB | 1.035 | 2.09E-08 | 1.10E-06 | 1.266 | 6.45E-11 | 9.47E-09 | 100144575_TGI_at | 0.370 | 7.43E-08 | 11719465_a_at | 0.438 | 1.19E-03 | 2.67E-02 | ++++ |
| Mr1 | MR1 | 0.164 | 1.60E-03 | 8.77E-03 | 0.235 | 1.92E-03 | 1.25E-02 | 100122336_TGI_at | 0.661 | 4.26E-13 | 11761914_a_at | 0.280 | 1.23E-02 | 9.60E-02 | ++++ |
| Atp6v0b | ATP6V0B | 0.144 | 1.93E-03 | 1.01E-02 | 0.335 | 1.41E-06 | 3.75E-05 | 100136768_TGI_at | 0.491 | 1.27E-13 | 11720314_s_at | 0.245 | 2.89E-05 | 3.38E-03 | ++++ |
| Atp6v0b | ATP6V0B | 0.144 | 1.93E-03 | 1.01E-02 | 0.335 | 1.41E-06 | 3.75E-05 | 100136768_TGI_at | 0.491 | 1.27E-13 | 200078_PM_s_at | 0.252 | 5.05E-05 | 4.61E-03 | ++++ |
| Atp6v0b | ATP6V0B | 0.144 | 1.93E-03 | 1.01E-02 | 0.335 | 1.41E-06 | 3.75E-05 | 100136768_TGI_at | 0.491 | 1.27E-13 | 11720313_a_at | 0.247 | 1.64E-04 | 8.64E-03 | ++++ |
| Atp6v0b | ATP6V0B | 0.144 | 1.93E-03 | 1.01E-02 | 0.335 | 1.41E-06 | 3.75E-05 | 100136768_TGI_at | 0.491 | 1.27E-13 | 11720315_x_at | 0.256 | 3.35E-04 | 1.32E-02 | ++++ |
| Atp6v0b | ATP6V0B | 0.144 | 1.93E-03 | 1.01E-02 | 0.335 | 1.41E-06 | 3.75E-05 | 100136768_TGI_at | 0.491 | 1.27E-13 | 11754478_x_at | 0.228 | 7.08E-04 | 2.01E-02 | ++++ |
| Atp6v0b | ATP6V0B | 0.144 | 1.93E-03 | 1.01E-02 | 0.335 | 1.41E-06 | 3.75E-05 | 100136768_TGI_at | 0.491 | 1.27E-13 | 11740194_a_at | 0.223 | 8.63E-04 | 2.22E-02 | ++++ |
| Npl | NPL | 0.308 | 1.74E-02 | 5.83E-02 | 0.346 | 1.56E-04 | 1.73E-03 | 100125641_TGI_at | 0.914 | 1.28E-11 | 11759566_a_at | 0.661 | 2.77E-03 | 4.25E-02 | ++++ |
| Npl | NPL | 0.308 | 1.74E-02 | 5.83E-02 | 0.346 | 1.56E-04 | 1.73E-03 | 100125641_TGI_at | 0.914 | 1.28E-11 | 11746995_a_at | 0.641 | 7.61E-03 | 7.43E-02 | ++++ |
| Npl | NPL | 0.308 | 1.74E-02 | 5.83E-02 | 0.346 | 1.56E-04 | 1.73E-03 | 100135716_TGI_at | 0.877 | 1.36E-11 | 11759566_a_at | 0.661 | 2.77E-03 | 4.25E-02 | ++++ |
| Npl | NPL | 0.308 | 1.74E-02 | 5.83E-02 | 0.346 | 1.56E-04 | 1.73E-03 | 100135716_TGI_at | 0.877 | 1.36E-11 | 11746995_a_at | 0.641 | 7.61E-03 | 7.43E-02 | ++++ |
| Npl | NPL | 0.308 | 1.74E-02 | 5.83E-02 | 0.346 | 1.56E-04 | 1.73E-03 | 100152229_TGI_at | 0.840 | 1.84E-10 | 11759566_a_at | 0.661 | 2.77E-03 | 4.25E-02 | ++++ |
| Npl | NPL | 0.308 | 1.74E-02 | 5.83E-02 | 0.346 | 1.56E-04 | 1.73E-03 | 100152229_TGI_at | 0.840 | 1.84E-10 | 11746995_a_at | 0.641 | 7.61E-03 | 7.43E-02 | ++++ |
| Npl | NPL | 0.308 | 1.74E-02 | 5.83E-02 | 0.346 | 1.56E-04 | 1.73E-03 | 100308805_TGI_at | 0.906 | 7.39E-12 | 11759566_a_at | 0.661 | 2.77E-03 | 4.25E-02 | ++++ |
| Npl | NPL | 0.308 | 1.74E-02 | 5.83E-02 | 0.346 | 1.56E-04 | 1.73E-03 | 100308805_TGI_at | 0.906 | 7.39E-12 | 11746995_a_at | 0.641 | 7.61E-03 | 7.43E-02 | ++++ |
| Npl | NPL | 0.308 | 1.74E-02 | 5.83E-02 | 0.346 | 1.56E-04 | 1.73E-03 | 100309471_TGI_at | 0.898 | 1.46E-11 | 11759566_a_at | 0.661 | 2.77E-03 | 4.25E-02 | ++++ |
| Npl | NPL | 0.308 | 1.74E-02 | 5.83E-02 | 0.346 | 1.56E-04 | 1.73E-03 | 100309471_TGI_at | 0.898 | 1.46E-11 | 11746995_a_at | 0.641 | 7.61E-03 | 7.43E-02 | ++++ |
| Pik3ap1 | PIK3AP1 | 0.465 | 1.77E-09 | 1.59E-07 | 0.358 | 1.56E-03 | 1.05E-02 | 100159223_TGI_at | 0.434 | 4.80E-07 | 11719029_at | 0.301 | 3.84E-03 | 5.06E-02 | ++++ |
| Inpp5a | INPP5A | -0.255 | 3.21E-06 | 6.05E-05 | -0.273 | 8.07E-04 | 6.34E-03 | 100122276_TGI_at | -0.320 | 1.34E-12 | 11719983_a_at | -0.327 | 5.31E-05 | 4.74E-03 | ---- |
| Inpp5a | INPP5A | -0.255 | 3.21E-06 | 6.05E-05 | -0.273 | 8.07E-04 | 6.34E-03 | 100122276_TGI_at | -0.320 | 1.34E-12 | 11719982_a_at | -0.283 | 1.99E-04 | 9.63E-03 | ---- |
| Inpp5a | INPP5A | -0.255 | 3.21E-06 | 6.05E-05 | -0.273 | 8.07E-04 | 6.34E-03 | 100122276_TGI_at | -0.320 | 1.34E-12 | 11757812_s_at | -0.262 | 8.43E-04 | 2.20E-02 | ---- |
| Rgs10 | RGS10 | 0.342 | 5.19E-06 | 8.96E-05 | 0.480 | 1.46E-09 | 1.28E-07 | 100312994_TGI_at | 0.400 | 1.17E-10 | 11740375_a_at | 0.323 | 5.43E-03 | 6.11E-02 | ++++ |
| Slc15a3 | SLC15A3 | 0.812 | 3.59E-12 | 1.36E-09 | 0.924 | 7.71E-13 | 2.81E-10 | 100125225_TGI_at | 0.634 | 2.20E-12 | 11746309_a_at | 0.304 | 1.01E-02 | 8.66E-02 | ++++ |
| Slco2b1 | SLCO2B1 | 0.287 | 7.11E-06 | 1.15E-04 | 0.526 | 1.07E-06 | 3.11E-05 | 100313532_TGI_at | 0.673 | 6.52E-13 | 11749132_a_at | 0.379 | 7.25E-04 | 2.03E-02 | ++++ |
| Slco2b1 | SLCO2B1 | 0.287 | 7.11E-06 | 1.15E-04 | 0.526 | 1.07E-06 | 3.11E-05 | 100313532_TGI_at | 0.673 | 6.52E-13 | 11739053_a_at | 0.275 | 7.80E-03 | 7.51E-02 | ++++ |
| Slco2b1 | SLCO2B1 | 0.287 | 7.11E-06 | 1.15E-04 | 0.526 | 1.07E-06 | 3.11E-05 | 100313532_TGI_at | 0.673 | 6.52E-13 | 11739051_a_at | 0.291 | 8.51E-03 | 7.85E-02 | ++++ |
| Slco2b1 | SLCO2B1 | 0.287 | 7.11E-06 | 1.15E-04 | 0.526 | 1.07E-06 | 3.11E-05 | 100313532_TGI_at | 0.673 | 6.52E-13 | 11762070_a_at | 0.133 | 1.01E-02 | 8.65E-02 | ++++ |
| Taldo1 | TALDO1 | 0.230 | 9.00E-06 | 1.38E-04 | 0.255 | 1.11E-05 | 2.07E-04 | 100150286_TGI_at | 0.375 | 1.06E-12 | 11715447_at | 0.169 | 3.90E-03 | 5.09E-02 | ++++ |
| Slc6a12 | SLC6A12 | 0.363 | 2.58E-04 | 2.02E-03 | 0.592 | 2.88E-05 | 4.43E-04 | 100155451_TGI_at | 1.338 | 9.40E-15 | 11731647_a_at | 0.217 | 2.82E-03 | 4.29E-02 | ++++ |
| Slc6a12 | SLC6A12 | 0.363 | 2.58E-04 | 2.02E-03 | 0.592 | 2.88E-05 | 4.43E-04 | 100155451_TGI_at | 1.338 | 9.40E-15 | 11740495_x_at | 0.140 | 6.89E-03 | 7.00E-02 | ++++ |
| Ddhd1 | DDHD1 | 0.249 | 7.96E-07 | 2.03E-05 | 0.330 | 4.00E-07 | 1.39E-05 | 100152276_TGI_at | 0.433 | 7.51E-12 | 11727208_x_at | 0.222 | 5.09E-03 | 5.90E-02 | ++++ |
| Rps27l | RPS27L | 0.088 | 8.78E-03 | 3.40E-02 | 0.105 | 3.66E-03 | 2.09E-02 | 100122259_TGI_at | 0.486 | 1.14E-12 | 11744350_a_at | 0.138 | 8.38E-03 | 7.82E-02 | ++++ |
| Rps27l | RPS27L | 0.088 | 8.78E-03 | 3.40E-02 | 0.105 | 3.66E-03 | 2.09E-02 | 100304179_TGI_at | 0.419 | 1.79E-12 | 11744350_a_at | 0.138 | 8.38E-03 | 7.82E-02 | ++++ |
| Cyba | CYBA | 0.765 | 2.25E-10 | 3.41E-08 | 0.879 | 4.40E-12 | 1.11E-09 | 100133799_TGI_at | 0.558 | 6.86E-10 | 11743968_a_at | 0.270 | 5.40E-04 | 1.70E-02 | ++++ |
| Cotl1 | COTL1 | 0.703 | 2.25E-08 | 1.16E-06 | 0.692 | 5.77E-10 | 6.10E-08 | 100159125_TGI_at | 0.306 | 9.24E-08 | 11715524_a_at | 0.265 | 6.07E-03 | 6.52E-02 | ++++ |
| Evi2a | EVI2A | 0.661 | 1.21E-07 | 4.47E-06 | 0.895 | 3.79E-09 | 2.99E-07 | 100136433_TGI_at | 0.549 | 5.87E-08 | 11733841_a_at | 0.369 | 2.06E-03 | 3.59E-02 | ++++ |
| Evi2a | EVI2A | 0.661 | 1.21E-07 | 4.47E-06 | 0.895 | 3.79E-09 | 2.99E-07 | 100310698_TGI_at | 0.524 | 3.37E-08 | 11733841_a_at | 0.369 | 2.06E-03 | 3.59E-02 | ++++ |
| Hn1 | HN1 | 0.186 | 7.86E-05 | 7.76E-04 | 0.371 | 3.48E-08 | 1.90E-06 | 100313130_TGI_at | 0.489 | 6.99E-13 | 11717726_s_at | 0.336 | 4.37E-04 | 1.51E-02 | ++++ |
| Hn1 | HN1 | 0.186 | 7.86E-05 | 7.76E-04 | 0.371 | 3.48E-08 | 1.90E-06 | 100313130_TGI_at | 0.489 | 6.99E-13 | 11717727_s_at | 0.177 | 1.15E-02 | 9.26E-02 | ++++ |
| Gngt2 | GNGT2 | 0.437 | 1.53E-04 | 1.33E-03 | 0.190 | 2.19E-02 | 7.97E-02 | 100124455_TGI_at | 0.867 | 5.31E-15 | 11756877_a_at | 0.166 | 1.13E-02 | 9.18E-02 | ++++ |
| Gngt2 | GNGT2 | 0.437 | 1.53E-04 | 1.33E-03 | 0.190 | 2.19E-02 | 7.97E-02 | 100310084_TGI_at | 0.689 | 5.22E-14 | 11756877_a_at | 0.166 | 1.13E-02 | 9.18E-02 | ++++ |
| Man2b1 | MAN2B1 | 0.321 | 1.07E-08 | 6.77E-07 | 0.389 | 1.63E-07 | 6.74E-06 | 100157242_TGI_at | 0.622 | 1.74E-13 | 11757369_s_at | 0.374 | 5.61E-04 | 1.75E-02 | ++++ |
| Ifi30 | IFI30 | 0.502 | 4.64E-08 | 2.07E-06 | 0.587 | 2.00E-09 | 1.70E-07 | 100146669_TGI_at | 0.378 | 3.00E-12 | 11755606_x_at | 0.233 | 1.10E-02 | 9.04E-02 | ++++ |
| Mcoln1 | MCOLN1 | 0.142 | 1.06E-03 | 6.31E-03 | 0.285 | 8.47E-06 | 1.65E-04 | 100150426_TGI_at | 0.615 | 4.58E-13 | 11748969_a_at | 0.410 | 5.80E-06 | 1.39E-03 | ++++ |
| Mcoln1 | MCOLN1 | 0.142 | 1.06E-03 | 6.31E-03 | 0.285 | 8.47E-06 | 1.65E-04 | 100150426_TGI_at | 0.615 | 4.58E-13 | 11718554_a_at | 0.314 | 3.45E-03 | 4.79E-02 | ++++ |
| Siglece | SIGLEC7 | 0.179 | 2.83E-03 | 1.37E-02 | 0.169 | 5.92E-03 | 3.03E-02 | 100141684_TGI_at | 0.898 | 1.81E-13 | 11733691_a_at | 0.393 | 6.22E-03 | 6.59E-02 | ++++ |
| Siglece | SIGLEC9 | 0.179 | 2.83E-03 | 1.37E-02 | 0.169 | 5.92E-03 | 3.03E-02 | 100141864_TGI_at | 0.899 | 2.41E-12 | 11729874_at | 0.369 | 2.78E-05 | 3.38E-03 | ++++ |
| Cyp1b1 | CYP1B1 | 2.431 | 7.16E-13 | 4.04E-10 | 0.887 | 9.33E-11 | 1.28E-08 | 100125484_TGI_at | 2.178 | 1.14E-19 | 11720411_s_at | 0.982 | 2.97E-09 | 3.27E-05 | ++++ |
| Cyp1b1 | CYP1B1 | 2.431 | 7.16E-13 | 4.04E-10 | 0.887 | 9.33E-11 | 1.28E-08 | 100125484_TGI_at | 2.178 | 1.14E-19 | 11720410_s_at | 0.942 | 4.87E-09 | 3.48E-05 | ++++ |
| Cyp1b1 | CYP1B1 | 2.431 | 7.16E-13 | 4.04E-10 | 0.887 | 9.33E-11 | 1.28E-08 | 100125484_TGI_at | 2.178 | 1.14E-19 | 11720409_s_at | 0.949 | 2.16E-08 | 8.21E-05 | ++++ |
| Cyp1b1 | CYP1B1 | 2.431 | 7.16E-13 | 4.04E-10 | 0.887 | 9.33E-11 | 1.28E-08 | 100125484_TGI_at | 2.178 | 1.14E-19 | 11747104_s_at | 0.868 | 5.87E-06 | 1.40E-03 | ++++ |
| Cyp1b1 | CYP1B1 | 2.431 | 7.16E-13 | 4.04E-10 | 0.887 | 9.33E-11 | 1.28E-08 | 100131143_TGI_at | 2.002 | 6.12E-20 | 11720411_s_at | 0.982 | 2.97E-09 | 3.27E-05 | ++++ |
| Cyp1b1 | CYP1B1 | 2.431 | 7.16E-13 | 4.04E-10 | 0.887 | 9.33E-11 | 1.28E-08 | 100131143_TGI_at | 2.002 | 6.12E-20 | 11720410_s_at | 0.942 | 4.87E-09 | 3.48E-05 | ++++ |
| Cyp1b1 | CYP1B1 | 2.431 | 7.16E-13 | 4.04E-10 | 0.887 | 9.33E-11 | 1.28E-08 | 100131143_TGI_at | 2.002 | 6.12E-20 | 11720409_s_at | 0.949 | 2.16E-08 | 8.21E-05 | ++++ |
| Cyp1b1 | CYP1B1 | 2.431 | 7.16E-13 | 4.04E-10 | 0.887 | 9.33E-11 | 1.28E-08 | 100131143_TGI_at | 2.002 | 6.12E-20 | 11747104_s_at | 0.868 | 5.87E-06 | 1.40E-03 | ++++ |
| Cyp1b1 | CYP1B1 | 2.431 | 7.16E-13 | 4.04E-10 | 0.887 | 9.33E-11 | 1.28E-08 | 100303658_TGI_at | 2.143 | 6.69E-20 | 11720411_s_at | 0.982 | 2.97E-09 | 3.27E-05 | ++++ |
| Cyp1b1 | CYP1B1 | 2.431 | 7.16E-13 | 4.04E-10 | 0.887 | 9.33E-11 | 1.28E-08 | 100303658_TGI_at | 2.143 | 6.69E-20 | 11720410_s_at | 0.942 | 4.87E-09 | 3.48E-05 | ++++ |
| Cyp1b1 | CYP1B1 | 2.431 | 7.16E-13 | 4.04E-10 | 0.887 | 9.33E-11 | 1.28E-08 | 100303658_TGI_at | 2.143 | 6.69E-20 | 11720409_s_at | 0.949 | 2.16E-08 | 8.21E-05 | ++++ |
| Cyp1b1 | CYP1B1 | 2.431 | 7.16E-13 | 4.04E-10 | 0.887 | 9.33E-11 | 1.28E-08 | 100303658_TGI_at | 2.143 | 6.69E-20 | 11747104_s_at | 0.868 | 5.87E-06 | 1.40E-03 | ++++ |
| Meis1 | MEIS1 | -0.293 | 2.50E-07 | 7.99E-06 | -0.275 | 6.62E-05 | 8.48E-04 | 100131820_TGI_at | -0.371 | 2.04E-10 | 11747255_a_at | -0.393 | 1.16E-03 | 2.63E-02 | ---- |
| Kynu | KYNU | 0.540 | 4.09E-06 | 7.42E-05 | 1.174 | 2.05E-12 | 5.69E-10 | 100125041_TGI_at | 0.815 | 4.47E-10 | 11719120_a_at | 0.592 | 4.54E-05 | 4.37E-03 | ++++ |
| Kynu | KYNU | 0.540 | 4.09E-06 | 7.42E-05 | 1.174 | 2.05E-12 | 5.69E-10 | 100125041_TGI_at | 0.815 | 4.47E-10 | 11733646_x_at | 0.310 | 5.23E-04 | 1.67E-02 | ++++ |
| Kynu | KYNU | 0.540 | 4.09E-06 | 7.42E-05 | 1.174 | 2.05E-12 | 5.69E-10 | 100302774_TGI_at | 0.953 | 2.80E-11 | 11719120_a_at | 0.592 | 4.54E-05 | 4.37E-03 | ++++ |
| Kynu | KYNU | 0.540 | 4.09E-06 | 7.42E-05 | 1.174 | 2.05E-12 | 5.69E-10 | 100302774_TGI_at | 0.953 | 2.80E-11 | 11733646_x_at | 0.310 | 5.23E-04 | 1.67E-02 | ++++ |
| Tm9sf4 | TM9SF4 | 0.138 | 3.01E-04 | 2.29E-03 | 0.204 | 2.77E-04 | 2.71E-03 | 100161017_TGI_at | 0.380 | 6.48E-09 | 11729576_a_at | 0.257 | 5.98E-04 | 1.82E-02 | ++++ |
| Tm9sf4 | TM9SF4 | 0.138 | 3.01E-04 | 2.29E-03 | 0.204 | 2.77E-04 | 2.71E-03 | 100161017_TGI_at | 0.380 | 6.48E-09 | 11747655_a_at | 0.197 | 6.17E-04 | 1.85E-02 | ++++ |
| Abcg1 | ABCG1 | 0.449 | 2.09E-07 | 6.93E-06 | 0.370 | 7.68E-05 | 9.53E-04 | 100157349_TGI_at | 0.301 | 3.88E-09 | 11725516_s_at | 0.390 | 4.60E-04 | 1.56E-02 | ++++ |
| Abcg1 | ABCG1 | 0.449 | 2.09E-07 | 6.93E-06 | 0.370 | 7.68E-05 | 9.53E-04 | 100157349_TGI_at | 0.301 | 3.88E-09 | 11750659_a_at | 0.432 | 6.77E-04 | 1.95E-02 | ++++ |
| Abcg1 | ABCG1 | 0.449 | 2.09E-07 | 6.93E-06 | 0.370 | 7.68E-05 | 9.53E-04 | 100157349_TGI_at | 0.301 | 3.88E-09 | 11725517_x_at | 0.408 | 8.61E-04 | 2.22E-02 | ++++ |
| Abcg1 | ABCG1 | 0.449 | 2.09E-07 | 6.93E-06 | 0.370 | 7.68E-05 | 9.53E-04 | 100157349_TGI_at | 0.301 | 3.88E-09 | 11725515_a_at | 0.379 | 1.90E-03 | 3.42E-02 | ++++ |
| Abcg1 | ABCG1 | 0.449 | 2.09E-07 | 6.93E-06 | 0.370 | 7.68E-05 | 9.53E-04 | 100157349_TGI_at | 0.301 | 3.88E-09 | 11726492_a_at | 0.327 | 1.23E-02 | 9.60E-02 | ++++ |
| Parvg | PARVG | 0.389 | 5.44E-07 | 1.50E-05 | 0.561 | 1.25E-07 | 5.50E-06 | 100140896_TGI_at | 0.629 | 2.06E-12 | 11740302_a_at | 0.283 | 1.15E-02 | 9.27E-02 | ++++ |
| Naga | NAGA | 0.173 | 9.88E-04 | 5.96E-03 | 0.292 | 2.90E-07 | 1.07E-05 | 100135929_TGI_at | 0.356 | 7.82E-10 | 11718450_at | 0.288 | 4.52E-04 | 1.55E-02 | ++++ |
| Naga | NAGA | 0.173 | 9.88E-04 | 5.96E-03 | 0.292 | 2.90E-07 | 1.07E-05 | 100135929_TGI_at | 0.356 | 7.82E-10 | 11748188_a_at | 0.211 | 8.98E-03 | 8.10E-02 | ++++ |
| Naga | NAGA | 0.173 | 9.88E-04 | 5.96E-03 | 0.292 | 2.90E-07 | 1.07E-05 | 100151247_TGI_at | 0.476 | 3.18E-10 | 11718450_at | 0.288 | 4.52E-04 | 1.55E-02 | ++++ |
| Naga | NAGA | 0.173 | 9.88E-04 | 5.96E-03 | 0.292 | 2.90E-07 | 1.07E-05 | 100151247_TGI_at | 0.476 | 3.18E-10 | 11748188_a_at | 0.211 | 8.98E-03 | 8.10E-02 | ++++ |
| Arl8b | ARL8B | 0.207 | 1.32E-05 | 1.86E-04 | 0.227 | 3.61E-05 | 5.29E-04 | 100305266_TGI_at | 0.392 | 1.09E-11 | 11721801_at | -0.142 | 7.73E-03 | 7.49E-02 | +++- |
| Cmtm7 | CMTM7 | 0.183 | 8.33E-04 | 5.26E-03 | 0.260 | 4.05E-04 | 3.63E-03 | 100143658_TGI_at | 0.528 | 6.56E-12 | 11721702_a_at | 0.394 | 2.64E-06 | 9.31E-04 | ++++ |
| Sparcl1 | SPARCL1 | -0.180 | 5.99E-04 | 4.03E-03 | -0.313 | 2.19E-03 | 1.38E-02 | 100142027_TGI_at | -0.377 | 3.98E-11 | 11730298_a_at | -0.329 | 3.78E-04 | 1.42E-02 | ---- |
| Sparcl1 | SPARCL1 | -0.180 | 5.99E-04 | 4.03E-03 | -0.313 | 2.19E-03 | 1.38E-02 | 100142027_TGI_at | -0.377 | 3.98E-11 | 11725023_a_at | -0.300 | 3.87E-04 | 1.43E-02 | ---- |
| Sparcl1 | SPARCL1 | -0.180 | 5.99E-04 | 4.03E-03 | -0.313 | 2.19E-03 | 1.38E-02 | 100142027_TGI_at | -0.377 | 3.98E-11 | 11752251_a_at | -0.322 | 4.56E-04 | 1.56E-02 | ---- |
| Dab2 | DAB2 | 0.340 | 1.59E-07 | 5.54E-06 | 0.478 | 7.73E-10 | 7.93E-08 | 100310184_TGI_at | 0.528 | 1.46E-11 | 11744797_s_at | 0.244 | 3.54E-03 | 4.86E-02 | ++++ |
| Dab2 | DAB2 | 0.340 | 1.59E-07 | 5.54E-06 | 0.478 | 7.73E-10 | 7.93E-08 | 100310184_TGI_at | 0.528 | 1.46E-11 | 11744796_a_at | 0.290 | 3.73E-03 | 4.97E-02 | ++++ |
| Dab2 | DAB2 | 0.340 | 1.59E-07 | 5.54E-06 | 0.478 | 7.73E-10 | 7.93E-08 | 100310184_TGI_at | 0.528 | 1.46E-11 | 11722356_a_at | 0.256 | 4.92E-03 | 5.77E-02 | ++++ |
| Hk3 | HK3 | 0.336 | 6.07E-05 | 6.34E-04 | 0.781 | 5.85E-08 | 2.90E-06 | 100121633_TGI_at | 0.969 | 2.44E-13 | 11757186_a_at | 0.318 | 8.19E-03 | 7.72E-02 | ++++ |
| Ghr | GHR | -0.197 | 9.30E-05 | 8.88E-04 | -0.240 | 4.90E-04 | 4.25E-03 | 100160347_TGI_at | -0.515 | 3.22E-11 | 11728055_at | -0.326 | 1.01E-03 | 2.43E-02 | ---- |
| Pla2g7 | PLA2G7 | 0.944 | 6.03E-12 | 1.89E-09 | 0.714 | 9.43E-07 | 2.79E-05 | 100147073_TGI_at | 1.785 | 3.82E-12 | 11761674_a_at | -0.119 | 2.72E-03 | 4.21E-02 | +++- |
| Pla2g7 | PLA2G7 | 0.944 | 6.03E-12 | 1.89E-09 | 0.714 | 9.43E-07 | 2.79E-05 | 100147073_TGI_at | 1.785 | 3.82E-12 | 11728421_a_at | 1.038 | 2.78E-03 | 4.26E-02 | ++++ |
| Tbxas1 | TBXAS1 | 0.618 | 1.21E-09 | 1.18E-07 | 1.050 | 9.73E-13 | 3.22E-10 | 100132994_TGI_at | 0.591 | 1.74E-13 | 11721661_a_at | 0.281 | 1.09E-02 | 9.00E-02 | ++++ |
| Tbxas1 | TBXAS1 | 0.618 | 1.21E-09 | 1.18E-07 | 1.050 | 9.73E-13 | 3.22E-10 | 100303239_TGI_at | 0.571 | 2.59E-13 | 11721661_a_at | 0.281 | 1.09E-02 | 9.00E-02 | ++++ |
| Ctsb | CTSB | 0.480 | 8.39E-10 | 8.84E-08 | 0.613 | 1.22E-11 | 2.47E-09 | 100151754_TGI_at | 0.633 | 1.56E-11 | 11756947_a_at | 0.363 | 3.55E-04 | 1.36E-02 | ++++ |
| Ctsb | CTSB | 0.480 | 8.39E-10 | 8.84E-08 | 0.613 | 1.22E-11 | 2.47E-09 | 100151754_TGI_at | 0.633 | 1.56E-11 | 11716715_a_at | 0.397 | 4.05E-04 | 1.46E-02 | ++++ |
| Ctsb | CTSB | 0.480 | 8.39E-10 | 8.84E-08 | 0.613 | 1.22E-11 | 2.47E-09 | 100151754_TGI_at | 0.633 | 1.56E-11 | 11716716_x_at | 0.422 | 4.77E-04 | 1.59E-02 | ++++ |
| Ctsb | CTSB | 0.480 | 8.39E-10 | 8.84E-08 | 0.613 | 1.22E-11 | 2.47E-09 | 100151754_TGI_at | 0.633 | 1.56E-11 | 11754977_x_at | 0.421 | 6.18E-03 | 6.57E-02 | ++++ |
| Asph | ASPH | -0.081 | 1.90E-02 | 6.24E-02 | -0.115 | 1.34E-02 | 5.53E-02 | 100140280_TGI_at | 0.395 | 3.44E-08 | 11750180_x_at | -0.205 | 9.75E-03 | 8.45E-02 | --+- |
| Tnfrsf10b | TNFRSF10B | 0.097 | 2.85E-02 | 8.57E-02 | 0.205 | 2.91E-02 | 9.91E-02 | 100140429_TGI_at | 0.376 | 7.23E-11 | 11719681_a_at | 0.263 | 1.05E-02 | 8.80E-02 | ++++ |
| Tnfrsf10b | TNFRSF10B | 0.097 | 2.85E-02 | 8.57E-02 | 0.205 | 2.91E-02 | 9.91E-02 | 100153254_TGI_at | 0.851 | 1.16E-14 | 11719681_a_at | 0.263 | 1.05E-02 | 8.80E-02 | ++++ |
| Tnfrsf10b | TNFRSF10B | 0.097 | 2.85E-02 | 8.57E-02 | 0.205 | 2.91E-02 | 9.91E-02 | 100310228_TGI_at | 0.827 | 6.07E-14 | 11719681_a_at | 0.263 | 1.05E-02 | 8.80E-02 | ++++ |
| Nek6 | NEK6 | 0.570 | 9.69E-10 | 9.81E-08 | 0.708 | 5.95E-08 | 2.94E-06 | 100150108_TGI_at | 1.034 | 1.27E-16 | 11753405_a_at | 0.206 | 8.51E-03 | 7.85E-02 | ++++ |
| Nek6 | NEK6 | 0.570 | 9.69E-10 | 9.81E-08 | 0.708 | 5.95E-08 | 2.94E-06 | 100302312_TGI_at | 0.918 | 6.94E-15 | 11753405_a_at | 0.206 | 8.51E-03 | 7.85E-02 | ++++ |
| Nek6 | NEK6 | 0.570 | 9.69E-10 | 9.81E-08 | 0.708 | 5.95E-08 | 2.94E-06 | 100312786_TGI_at | 1.025 | 8.47E-17 | 11753405_a_at | 0.206 | 8.51E-03 | 7.85E-02 | ++++ |

logFC: Log fold change. FDR: false discovery rate

## Supplementary Table 2: Top 50 differentially expressed genes in lung tissue between mild COPD and controls from the lung eQTL study.

| **probesetid** | **estimate** | **SE** | **P value** | **FDR** | **Gene Symbol** | **GeneName** | |
| --- | --- | --- | --- | --- | --- | --- | --- |
| 100301279_TGI_at | -0.765 | 0.145 | 1.46E-07 | 0.008 | TBC1D20 | TBC1 domain family, member 20 | |
| 100157900_TGI_at | 0.686 | 0.138 | 6.95E-07 | 0.018 |  |  | |
| 100131620_TGI_at | -0.657 | 0.143 | 4.06E-06 | 0.048 |  |  | |
| 100300152_TGI_at | -0.625 | 0.136 | 4.10E-06 | 0.048 |  | DKFZP434B0335 protein | |
| 100305188_TGI_at | 0.616 | 0.135 | 4.86E-06 | 0.048 | MAPK6 | mitogen-activated protein kinase 6 | |
| 100151206_TGI_at | -0.657 | 0.145 | 5.63E-06 | 0.048 |  |  | |
| 100130091_TGI_at | -0.630 | 0.140 | 6.41E-06 | 0.048 | LGR5 | leucine-rich repeat-containing G protein-coupled receptor 5 | |
| 100310107_TGI_at | 0.695 | 0.157 | 9.03E-06 | 0.059 | HPDL | 4-hydroxyphenylpyruvate dioxygenase-like | |
| 100141758_TGI_at | -0.615 | 0.140 | 1.12E-05 | 0.065 |  |  | |
| 100130887_TGI_at | 0.591 | 0.135 | 1.24E-05 | 0.065 | MAPK6 | mitogen-activated protein kinase 6 | |
| 100305135_TGI_at | 0.641 | 0.149 | 1.58E-05 | 0.066 | MOBKL3 | MOB1, Mps One Binder kinase activator-like 3 (yeast) | |
| 100153167_TGI_at | 0.589 | 0.137 | 1.61E-05 | 0.066 | SMOX | spermine oxidase | |
| 100157304_TGI_at | -0.654 | 0.152 | 1.64E-05 | 0.066 |  | hypothetical protein FLJ39639 | |
| 100125289_TGI_at | 0.639 | 0.150 | 2.13E-05 | 0.080 |  |  | |
| 100139748_TGI_at | 0.649811 | 0.154432 | 2.58E-05 | 0.090062 | AB073349 |  | |
| 100147253_TGI_at | 0.575699 | 0.137996 | 3.02E-05 | 0.098906 | AA040195 | PANX1 | pannexin 1 |

SE: standard error. FDR: false discovery rate

## Supplementary Table 3: Associations of eQTLs with lung function measures in large scale genetic data. Shown are SNPs with P<0.05

| SNP | geneSymbol | Phenotype | p.value | FDR |
| --- | --- | --- | --- | --- |
| rs1081512 | CTSS | SpiroMeta 1000G FEV1 | 6.07E-05 | 4.43E-03 |
| rs140113454 | NQO1 | UKBiLeve Lo vs. Hi FEV1 Never Smoker | 3.85E-03 | 2.73E-01 |
| rs1081512 | CTSS | UKBiLeve Lo vs. Hi FEV1 Never Smoker | 4.29E-03 | 3.00E-01 |
| rs7873147 | NEK6 | UKBiLeve Lo vs. Hi FEV1 Never Smoker | 5.91E-03 | 4.08E-01 |
| rs1641546 | CD68 | UKBiLeve Lo vs. Hi FEV1 Never Smoker | 6.15E-03 | 4.18E-01 |
| rs499606 | NEU1 | UKBiLeve Lo vs. Hi FEV1 Never Smoker | 9.52E-03 | 6.38E-01 |
| rs145123180 | ITGAM | UKBiLeve Lo vs. Hi FEV1 Heavy Smoker | 1.26E-02 | 8.95E-01 |
| rs1081512 | CTSS | UKBiLeve Lo vs. Hi FEV1 Heavy Smoker | 1.36E-02 | 9.52E-01 |
| rs728624 | LRRK2 | UKBiLeve Lo vs. Hi FEV1 Never Smoker | 1.44E-02 | 9.50E-01 |
| rs153444 | GM2A | UKBiLeve Lo vs. Hi FEV1 Never Smoker | 1.68E-02 | 1 |
| rs2070902 | FCER1G | SpiroMeta 1000G FEV1FVC | 2.22E-02 | 1 |
| rs10089616 | CLN8 | SpiroMeta 1000G FEV1 | 2.23E-02 | 1 |
| rs7905087 | PI4K2A | UKBiLeve Lo vs. Hi FEV1 Never Smoker | 2.33E-02 | 1 |
| rs7084521 | PI4K2A | UKBiLeve Lo vs. Hi FEV1 Never Smoker | 2.40E-02 | 1 |
| rs2847341 | PGD | UKBiLeve Lo vs. Hi FEV1 Never Smoker | 2.48E-02 | 1 |
| rs7681694 | SPARCL1 | SpiroMeta 1000G FEV1FVC | 2.65E-02 | 1 |
| rs12902921 | RPS27L | UKBiLeve Lo vs. Hi FEV1 Never Smoker | 2.68E-02 | 1 |
| rs760456 | ITGB2 | SpiroMeta 1000G FEV1 | 2.71E-02 | 1 |
| rs760457 | ITGB2 | SpiroMeta 1000G FEV1 | 2.86E-02 | 1 |
| rs6428945 | GPR137B | SpiroMeta 1000G FEV1 | 3.27E-02 | 1 |
| rs1641546 | CD68 | SpiroMeta 1000G FEV1 | 3.52E-02 | 1 |
| rs7223589 | EVI2A | UKBiLeve Lo vs. Hi FEV1 Never Smoker | 3.56E-02 | 1 |
| rs6428945 | GPR137B | SpiroMeta 1000G FEV1FVC | 3.77E-02 | 1 |
| rs144069380 | CAPG | SpiroMeta 1000G FEV1FVC | 4.22E-02 | 1 |
| rs12983058 | SIGLEC7 | UKBiLeve Lo vs. Hi FEV1 Heavy Smoker | 4.22E-02 | 1 |
| rs7223589 | EVI2A | SpiroMeta 1000G FEV1FVC | 4.40E-02 | 1 |
| rs353633 | CD44 | UKBiLeve Lo vs. Hi FEV1 Heavy Smoker | 4.76E-02 | 1 |
